# Overexpression of *CERKL*, a gene responsible for retinitis pigmentosa in humans, protects cells from apoptosis induced by oxidative stress

**Published:** 2009-01-21

**Authors:** Miquel Tuson, Alejandro Garanto, Roser Gonzàlez-Duarte, Gemma Marfany

**Affiliations:** 1 Departament de Genètica, Facultat de Biologia, Universitat de Barcelona, Barcelona, Spain; 2 Institut de Biomedicina de la Universitat de Barcelona (IBUB), Barcelona, Spain; 3 Centre for Biomedical Research on Rare Diseases (CIBER-ER), Instituto de Salud Carlos III, Barcelona, Spain

## Abstract

**Purpose:**

Retinitis pigmentosa (RP), a retinal neurodegenerative disorder characterized by apoptosis of photoreceptor cells, is caused by mutations in many different genes. We analyzed the RP gene ceramide kinase-like (*CERKL*) to determine CERKL function and contribution to pathogenesis.

**Methods:**

RT–PCR was performed to characterize *CERKL* expression in many human adult and fetal tissues, including retina. We analyzed the protein subcellular localization by confocal microscopy and further verified it by sucrose gradients. We performed lipid kinase activity assays. And finally, we studied the effects on cell apoptosis after *CERKL* overexpression in transiently transfected cultured cells by propidium iodide staining and poly-(ADP-ribose)-polymerase (PARP) caspase-dependent cleavage.

**Results:**

*CERKL* transcripts underwent alternative splicing. In the human retina, four different CERKL isoforms of 532, 558, 419, and 463 amino acids were expressed. CERKL proteins were mainly localized in the endoplasmic reticulum and Golgi compartments, but they also shifted localization to nuclei and nucleoli. We also found that CERKL prevented cells from entering apoptosis induced by oxidative-stress conditions.

**Conclusions:**

CERKL remains a unique orphan lipid kinase in that no candidate substrate has been identified after intense research. The dynamic localization of CERKL suggests multiple sites of action. Remarkably, CERKL (but not the RP R257X mutant) exerts a protective role in cells against oxidative stress, consistent with RP mutations impairing the normal protein function in photoreceptors and thus tilting the balance toward apoptosis. These results provide valuable insights into the molecular mechanisms causing retinal degeneration.

## Introduction

Retinitis pigmentosa (RP) is an inherited retinal neurodegenerative disorder characterized by the progressive attrition of photoreceptor cells through apoptosis. Although mutations in many different genes have the same phenotypic outcome, there is not much information gathered on the biochemical processes linking the genotype to the eventual photoreceptor death. In the course of our search for new genes causing autosomal recessive RP in several Spanish families, we identified a previously unannotated gene, ceramide-kinase like (*CERKL*) [1,2]. Mutations in *CERKL* are responsible for a phenotypically distinct RP [2,3], with characteristic macular and peripheral lesions. The name of the gene stems from the diacylglycerol kinase domain–which shares homology to ceramide kinases–of the encoded protein.

Ceramide is a chore sphingolipid (SL), being the precursor of other bioactive and complex SLs and one of the initial key players in stress-induced apoptosis [4]. SLs are now considered lipid second messengers that behave as finely tuned sensors of cell status [4,5]. Accordingly, the enzymes involved in SL metabolism, and among them CERKL, are rheostats that integrate multiple stress stimuli [6]. Mutations in these genes cause multiple and complex phenotypic effects, including severe neurodegenerative disorders [7]. A variety of extracellular and intracellular stimuli, including cytokines, cytotoxic agents, and stress signals, cause the accumulation of ceramide via sphingomyelin hydrolysis or de novo synthesis. To avoid entering apoptosis, cells counteract this ceramide increase by activating enzymatic pathways involved in ceramide clearance. In this context, the phosphorylation of ceramide by ceramide kinase exerts a protective role against apoptosis. Ceramide can be produced in different subcellular membranous compartments, adding to the complexity and the homeostasis of the whole system [6,8]. Therefore, the identification of *CERKL* as an RP gene provided a missing link between alterations in SL metabolism to inheritable retinal disorders and suggested a new avenue for elucidating the ethiopathological mechanisms.

Here we report that *CERKL* undergoes alternatively splicing events, which affect the exons encoding the lipid kinase domain. Four protein isoforms are generated, although only 2 of them contain the complete diacylglycerol (DAG) kinase domain. Here we present a detailed characterization of the expression of these 4 alternatively spliced isoforms in different human tissues, and demonstrate that these 4 variants are only present in the retina. The 4 proteins show a similar subcellular localization pattern, and are mainly located in the ER and Golgi compartments. Although *CERKL* substrates remains elusive under standard experimental conditions, we show that overexpression of CERKL isoforms protects cells from apoptosis induced by oxidative stress.

## Methods

### Cloning of *CERKL* splicing variants in the human retina

Specific primers flanking exons 2 and 13 (Table 1) were used to clone *CERKL* alternative splicing variants by PCR from Marathon-Ready™ human retina cDNA (BD Biosciences, Franklin Lakes, NJ) under the following conditions: 94 °C for 30 s, 58 °C for 30 s, and 72 °C for 2 min for 35 cycles. The primer CERKL_E2_F annealed to exon 2 while the CERKL_E13_R primer annealed to exon 13 and introduced a BamHI site to facilitate subsequent subcloning. The 50 μl reaction mixture contained 10 μM of each primer, 2 μM of dNTPs, 1.5 mM MgCl_2_ and 1 U of Pfu Turbo polymerase (Stratagene, La Jolla, CA). The PCR bands detected after electrophoresis on a 1.5% agarose gel were cloned into the EcoRV site of pBluescriptII KS and verified by sequencing. The full-length cDNAs of these *CERKL* isoforms were reconstituted by digesting the partial clones with EcoRI and BamHI and subcloning the corresponding fragments into a pBluescriptII KS vector that already contained the 5′ end of *CERKL* (the complete exon 1 and the stretch of exon 2 before the EcoRI site, obtained from the IMAGE clone 3870103).

**Table 1:** CERKL amplification primers for the construction of expression vectors and the RT–PCR analyses.

**Forward primers (5′-3′)**	
**CERKL_E2_F**	CTGTTAAACAGCAGAGAAGTGGTAC
**RT_CERKLab_F**	GTAACAATAATGGAATATGAAGGG
**RT_CERKLc_F**	CAGTTCAAGAAAATATTGGCAGGATC
**RT_CERKLd_F**	GGAATAAAAACTGATGTAACAAGATC
**mutCERKL_F^c^**	TGGGATGGAAACAGAC**T**GAATCCTGACTCCTGTC
**Reverse primers (5′-3′)**	
**CERKL_E13_R^a^**	AT*GGATCC*TTACTTTGGAATCATTTCTTCCATG
**RT_CERKLab_R**	ATCCTGCTGGTATTAAGCCAAG
**RT_CERKLcd_R**	CCTTAACAACAGCAAAATCTCTCCG
**CERKL_E13_HA_R^a,b^**	CG*GATATC*AAGCGTAATCTGGAACATCGTATGGGTACTTTGGAATCATTTCTTCC
**mutCERKL_R^c^**	GACAGGAGTCAGGATTC**A**GTCTGTTTCCATCCCA

^a^CERKL_E13_R and CERKL_E13_HA_R introduce a restriction site (indicated in italics) for subsequent cloning. ^b^CERKL_E13_HA_R contains the coding sequence of the HA epitope (underlined). ^c^The truncated R257X CERKL proteins were obtained by site-directed mutagenesis using the primers mutCERKL_F and mutCERKL_R, which introduce the 847C>T change (in bold and underlined).

### RT–PCR analysis of the presence of *CERKL* splicing variants in human tissues

The splicing pattern of *CERKL* was analyzed in human retina cDNA and a panel of first-strand cDNAs from several human adult and fetal tissues, using specifically designed pairs of primers to amplify and distinguish each isoform (Table 1), as follows: 1) to detect splicing variants *CERKLa* and *CERKLb* (RT_CERKLab_F and RT_CERKLab_R); 2) to amplify splicing variant *CERKLc* (RT_CERKLc_F and RT_CERKLcd_R); 3) to detect splicing variant *CERKLd* (RT_CERKLd_F and RT_CERKLcd_R). Two-step PCR conditions were as follows: 35 cycles of 94 °C for 30 s and 58 °C for 30 s. The 25 μl reaction mixture contained 10 μM of each primer, 2 μM of dNTPs, 1.5 mM MgCl_2_ and 1 U Taq polymerase (Promega, Madison, WI). The concentration of the Marathon-Ready™ human retina cDNA and first-strand cDNAs from the tissue panel (BD Biosciences) in the reactions were 0.01 ng/μl and 0.1 ng/μl, respectively.

### Overexpression of *CERKL* isoforms in cultured cells and preparation of protein cell lysates

Expression vectors containing the full-length open reading frame of the four *CERKL* isoforms were assembled by PCR amplification of each variant using the CERKL_E2_F primer, which anneals upstream of the EcoRI site in exon 2 and the CERKL_E13_HA_R primer, which anneals in exon 13 (Table 1). Primer CERKL_E13_HA_R also encoded a C-terminal hemagglutinin (HA) epitope tag and included an EcoRV site for subsequent cloning. The PCR products were digested with EcoRI and EcoRV and cloned into a pcDNA3 (Invitrogen Life Technologies, Carlsbad, CA) that contained exons 1 and 2 of *CERKL* (previously cloned between HindIII and EcoRI sites). The integrity of all constructs was verified by sequencing. To generate the truncated RP mutant protein (R257X), site-directed mutagenesis was performed on the 2 constructs containing the isoforms CERKLa and CERKLb (encompassing the lipid kinase domain). The 847C>T change was introduced with the mutCERKL_F and mutCERKL_R primers (Table 1). In these constructs, the HA epitope was added to the N-terminus of the protein for immunodetection.

Human embryonic kidney (HEK293T) or African green monkey kidney (COS-7) cells were seeded at 3×10^6^ cells/dish in 10 cm cell culture dishes 1 day before transfection. Cells were tranfected with 24 µg/dish using either the pcDNA3 vector alone or each pcDNA3–CERKL construct and 60 µl/dish of Lipofectamine™2000 (Invitrogen Life Technologies), following the manufacturer’s instructions. After 2 days, the cells were harvested and lysed by sonication in lysis buffer [20 mM MOPS pH 7.2, 2 mM EGTA, 1 mM dithiothreitol, 10% glycerol, and complete™ protease inhibitor (Roche Diagnostics, Indianapolis, IN)], as described elsewhere [9].

### In vitro and in vivo kinase activity assays

DAG and ceramide kinase activities were assayed in vitro as described [10]. Briefly, the source of the enzymatic activity were protein lysates from either HEK293T or COS-7 cells transfected with each CERKL isoform (250 µg total protein), the empty vector or a CERK-expressing construct (negative and positive control, respectively) obtained by sonication in 20 mM MOPS pH 7.0, 2 mM EGTA, 1 mM DTT, 10% glycerol plus protease inhibitors. The source of lipids were either: a) micelles obtained from commercial sphingolipids, which contained 880 µM lipids (ceramide and sphingosine, both from bovine brain sphingomyelin, or 1,2-dioleoyl-sn-glycerol, Sigma-Aldrich, St. Louis, MO), 1 mM cardiolipin, 1.5% β-octylglucoside, 0.2 mM diethylenetriamine-pentaacetic acid (DETAPAC); b) lipid micelles, obtained by sonication of 50 μg of lipids extracted from cultured 661W murine photoreceptor-derived cells in 1 mM DETAPAC, 7.5% β-octylglucoside; or c) total 661W cell lysates that were heat-inactivated at 65 °C for 20 min to abrogate endogenous enzymatic activities. Reactions were performed at 100 µl final volume containing 20 mM MOPS pH 7.2, 50 mM NaCl, 1 mM dithiothreitol, 2 mM EGTA, and 3 mM CaCl_2_, with the corresponding lipid and protein sources. Reactions were started by adding MgCl_2_ at 0.5 mM final concentration plus γ-^32^P-ATP (2–5 µCi/reaction), incubated at 30 °C for 30 min, 60 min, or overnight, and stopped with 250 µl of chloroform, 250 µl of methanol and 125 µl HCl 2.4 M. After vortexing, the phases were separated by centrifugation, and the organic phase removed and vacuum-dried. Lipids were resuspended in 25 µl chloroform:methanol (95:5, v/v), spotted onto silica gel plates and separated by thin-layer chromatography (TLC). For the enzymatic assays in vivo, we seeded 1.5×10^6^ 661W cells on 10 cm diameter plates and transfected using either the two pEGP-CERKL long isoforms (a and b) or the pEGFP empty-vector (negative control). Transfected cells were enriched by using Fluorescence Activated Cell Sorting (FACS) to select green fluorescent protein (GFP) positive cells. These cells were reseeded on smaller plates, and 4 μl of radioactive ^32^P-orthophosphate was added to the medium. Cells were collected after 4 h, and lysed directly in a solution of chloroform:methanol:chlorhydric acid (100:100:1). After vigorous vortexing, cellular debris was sedimented by centrifugation in a microfuge (100x g) and the liquid phase was vacuum-dried on completion. The lipid pellet was resuspended in a solution of 1:1 chloroform:methanol and loaded onto silica gel 60 TLC plates (Merck, Whitehouse Station, NJ). All TLCs (assays in vivo and in vitro) were run in chloroform:acetone:methanol:acetic acid:water (10:4:3:2:1, v/v). Radiolabeled lipids were visualized by autoradiography.

### Purification of recombinant proteins

To express the four CERKL isoforms as glutathione-S-transferase (GST) fusion proteins, we subcloned each variant in-frame into pGEX-4T-1 (Amersham Biosciences, Chalfont St. Giles, UK), between the XhoI and NotI sites. The constructs were transformed into BL21 Codon Plus *E.coli* cells (Stratagene) to avoid premature protein truncation due to the strongly biased codon usage of the *CERKL* human gene. Recombinant proteins were produced in 500 ml cultures induced by the addition of 0.5 mM Isopropyl-β-D-thiogalactopyranoside (IPTG). Purified GST-recombinant proteins were obtained after elution with reduced glutathione of the protein bound to the glutathione-sepharose bead batch, following the manufacturer’s instructions (Amersham Biosciences).

### Lipid-protein overlay assay

Binding of CERKL isoforms to sphingolipids was assessed using SphingoStrips™ and PIP Strips™ (Echelon Biosciences, Salt Lake City, UT), which consist of several nitrocellulose-immobilized sphingolipids and phosphoinositides (100 pmol/spot) respectively. The membranes were equilibrated for 5 min in 10 mM Tris-HCl pH 8.0, 150 mM NaCl, and 0.1% v/v Tween-20 (TBST). They were then blocked with 3% BSA in TBST (blocking solution) for 1 h at room temperature. Subsequently, the membrane was then incubated overnight at 4 °C in blocking solution containing 0.5 µg/ml of the GST-fusion protein on a rocking platform. The membranes were washed twice for 15 min in TBST with gentle agitation, and then incubated for 1 h with 1:1,000 anti-GST monoclonal antibody (Santa Cruz Biotechnology, Santa Cruz, CA). After washing as before, membranes were incubated for 1 h with 1:3,000 horseradish peroxidase-conjugated anti-mouse IgG antibody (Sigma). Finally, the membranes were washed 6 times in an hour in TBST, and developed with the ECL western blotting detection system (Amersham Biosciences).

### Transfection, immunolocalization and confocal laser microscopy

COS-7 cells were grown in Dubecco’s Modified Eagle Medium (DMEM) containing 10% fetal bovine serum, 4 mM L-glutamine, 100 U/ml of penicillin, and 100 µg/ml streptomycin (Invitrogen Life Technologies). Cells were grown on coverslips in 12 well plates. Transient transfections were performed using Lipofectamine™2000 (Invitrogen Life Technologies) according to the manufacturer’s protocol. Next, 24 h or 48 h post-transfection cells were rinsed with PBS 1X (137 mM NaCl, 2.7 mM KCl, 1.5 mM KH_2_PO_4_, 8 mM Na_2_HPO_4_, pH 7.4) and fixed in 3% paraformaldehyde and 2% sucrose in 0.1 M phosphate buffer at 4 °C for 30 min. Cells were then washed and permeabilized for 10 min with a solution that contained 0.1% Triton X-100, 20 mM glycine, and 10 mM PBS. After permeabilization, cells were rinsed and blocked in a solution composed of 1% BSA, 20 mM glycine, and 10 mM PBS. Then, cells were incubated with 1:250 anti-HA polyclonal antibody and either specific 1:25 anti-calnexin, 1:250 anti-GM130, or 1:1,000 anti-EEA1 monoclonal primary antibodies (all from BD Biosciences) at 37 °C for 1 h. Mitochondria were labeled by adding reduced MitoTracker Orange (Molecular Probes, Invitrogen Life Technologies, Carlsbad, CA) to the cell culture medium at a final concentration of 500 nM for 45 min before the fixation procedure and subsequent immunocytochemistry. Upon washing, cells were incubated with 1:300 AlexaFluor 488-conjugated anti-rabbit and 1:300 AlexaFluor 546-conjugated anti-mouse (Molecular Probes) secondary antibodies. When required, slides were counter-stained with 1:200 DAPI (Sigma) nuclear blue dye in PBS for 15 min. All preparations were mounted in Vectashield medium for fluorescence (Vector Laboratories, Burlingame, CA) and analyzed by confocal laser scanning microscopy with Olympus Fluoview 500 (Olympus, Tokyo, Japan) and Leica TSC NT and TSC SPII (Leica, Wetzlar, Germany) laser scanning microscopes. In silico predictions for CERKL subcellular localization were performed using PSORT, ProSLP v2.0, ESLPred, and SubLoc v1.0.

### Subcellular fractionation by sucrose gradient

HeLa cells (human cervical cancer derived cell line ATCC CCL-2) were seeded (1.5×10^6^ cells/dish) in 10 cm plates and transfected with the CERKLa isoform tagged with the HA epitope. At 48 h post-transfection, cells were washed twice with 1X PBS, and then incubated 20 min at 4 °C in a solution that contained 10 mM Tris HCl, pH 7.4, 3% sucrose w/v, and 1% protease inhibitors (Roche). After this incubation, the concentration of sucrose was increased up to 0.25 M, and cells were broken by 15 strokes in an iced-chilled Dounce homogenizer. Cellular debris, nuclei, and unbroken cells were separated by centrifugation at 1,300x g for 5 min at 4 °C, and the supernatant was further centrifuged overnight at 100,000x g at 4 °C. The pellet containing cell organelles was resuspended in 1.5 M sucrose and laid at the bottom of a 1.8 ml swing-out (Beckman, Fullerton, CA) centrifuge tube. Subsequent layers of equal volumes of 1.15, 0.9, 0.6, and 0.25 M sucrose were added, and the final sucrose gradient was centrifuged for 2 h at 100,000x g at 4 °C. Next, 11 fractions of approximately 150 μl were collected from bottom to top. Samples were loaded onto 10% polyacrylamide SDS–PAGE gels, transferred to 0.45 µm polyvinylidene fluoride PDVP membranes (Immobilon-P, Millipore, Billerica, MA) and underwent immunodetection with the corresponding antibodies.

### Immunodetection

After blocking membranes for 1 h in 10% nonfat milk in phosphate buffered saline, 0.1% Tween-20 (MTP), membranes were incubated overnight at 4 °C or 1 h at room temperature, separately, with any of the following primary antibodies, all diluted at 1:1000, anti-GM130 (BD Biosciences), anti-PDI (BD Biosciences), anti human TGN38 (Santa Cruz Biotechnology, Inc.) or anti-HA (Covance, Princetown, NJ). After extensive washes in MTP, membranes were incubated for 1 h with 1:3,000 of the corresponding peroxidase-conjugated secondary antibody (Sigma), and the bands were visualized using the ECL western blotting detection system (Amersham Biosciences).

### Propidium iodide staining assay

COS-7 cells were grown on coverslips in 24 well plates. Transient transfections were performed on the following: the constructs encoding each of the four isoforms (pcDNA3-CERKL), the truncated variant (pcDNA3-R257X), empty pcDNA3.1 (negative control), or pcDNA6.2-DEST-CERK (provided by F. Bornancin, Novartis). At 44 h post-transfection, cells were treated with 200 µM H_2_O_2_ (Fluka, Sigma-Aldrich, St. Louis, MO) and 3 h later propidium iodide was added at a final concentration of 7 µg/ml. After 1 h, cells were rinsed twice with PBS and fixed in 3% paraformaldehyde and 2% sucrose in 0.1 M phosphate buffer at 4 °C for 30 min. They were then washed 2 times in 0.1 M PBS and mounted in Vectashield medium (Vector Laboratories). Several fields were analyzed under the visible and green light channels in a fluorescence microscope (Leica DM IL) coupled to a Leica DFC camera (Leica, Wetzlar, Germmany). A minimum of 500 cells were counted and compared to detect pignotic nuclei in the total cell count. Three independent replicates were performed for each construct.

### Analysis of PARP cleavage-dependent apoptosis

HeLa cells were seeded in 24 well plates and transfected with either empty pcDNA3.1 (negative control) or pcDNA3-CERKLa (containing the lipid kinase domain). At 40 h post-transfection, the medium was replaced by fresh solution containing one of the following: 300 µM H_2_O_2_, 400 µM H_2_O_2_, or 0.3 M sodium nitroprusside (Fluka), or serum-deprived medium. After 24 h, cells were rinsed with PBS, harvested, and lysed directly in protein electrophoresis loading buffer. Samples were then boiled for 5 min and centrifuged to sediment cellular debris. Protein preparations were loaded onto 7.5% SDS–PAGE gels. Either 1:1,000 monoclonal anti-PARP (Invitrogen Life Technologies) or 1:8,000 monoclonal anti-tubulin (Sigma) were used as primary antibodies. Anti-mouse secondary antibody was conjugated to 1:3,000 horseradish peroxidase. Immunodetection was performed using chemiluminescent ECL reagents (GE Healthcare, Waukesha, WI), and the intensity of each band was quantified using the Quantity-One 1-D Analysis Software (Bio-Rad, Hercules, CA).

## Results

### CERKL transcripts are alternatively spliced in the human retina

**Figure 1:**
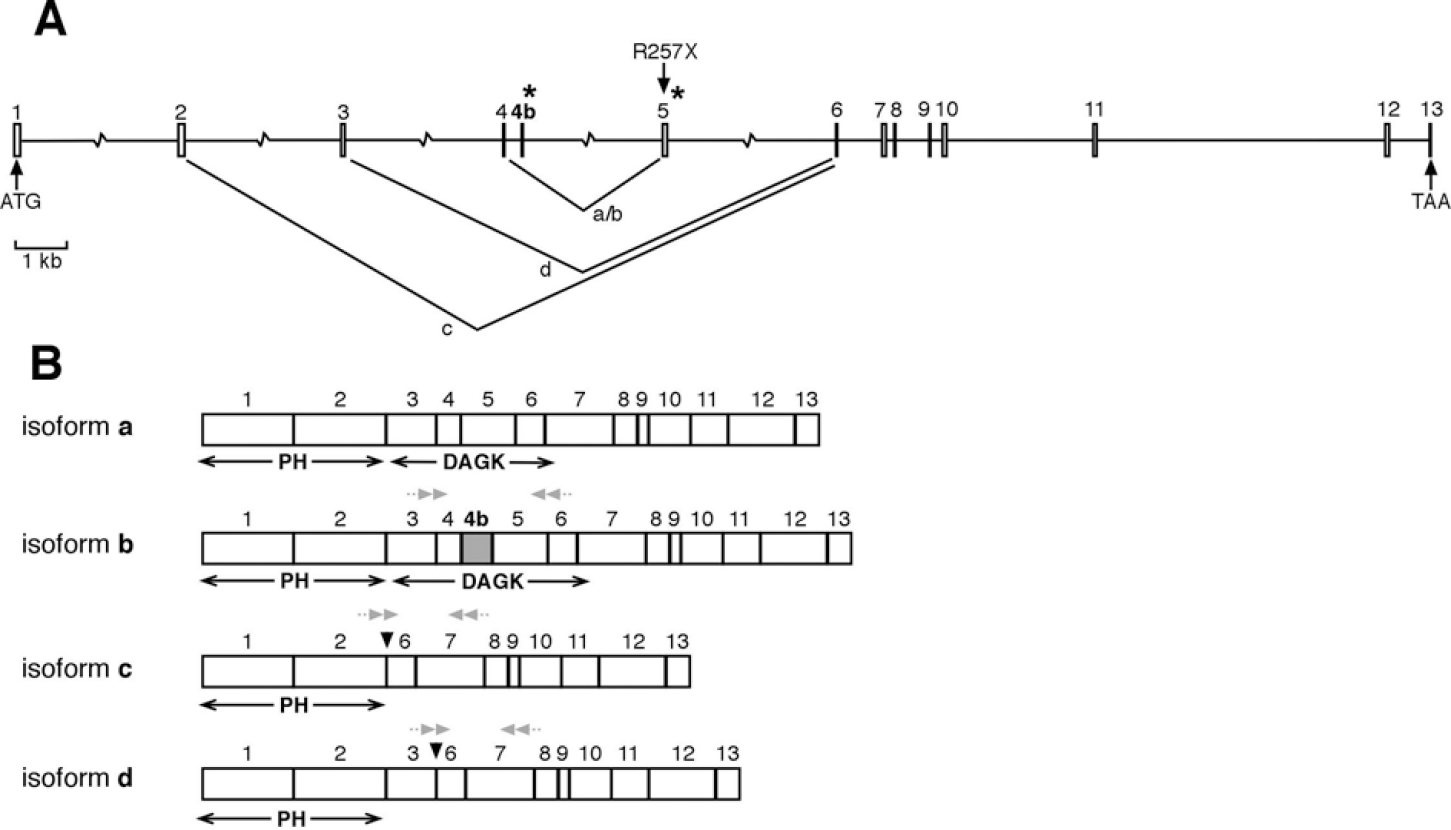
*CERKL* isoform structure. **A:** The diagram shows the genomic exon-intron structure of the *CERKL* gene with the initiation and stop codons. The position of the nonsense mutation (R257X) identified in RP families as well as the splicing junctions resulting in isoforms a, b, c, and d are indicated. Asterisks indicate the position of the rare GC-splicing donor sites. **B:** The mature *CERKL* mRNA isoforms, indicating the relative position of the encoded DAG kinase and putative PH domains, are depicted. Note that exon 4b (isoform b, in gray) is located well within the DAG kinase domain, while isoforms c and d lack this domain. Grey small double arrows indicate the position of the primers used for specific isoform RT–PCR analyses (see Methods). The figure is drawn to scale except for the introns depicted with a broken line.

The initially characterized *CERKL* gene spanned 13 exons in chromosome 2q31.2–31.3 (Figure 1A). While attempting to clone the full-length *CERKL* on retina cDNA using primers spanning the ATG (forward) and the STOP (reverse) codons, we observed several faint bands, which were indicative of alternatively spliced variants. Therefore, we devised a two-step strategy of RT–PCR and cloning (see Methods) to characterize the full-length isoforms based on the structure of the gene. The RT–PCR analysis of human retina mRNA using primers from exons 2 and 13 allowed us to identify several spliced transcripts (Figure 1A) that would result in several protein isoforms (Figure 1 B): 1) CERKLa, which corresponded to the already reported variant [2], spanning 13 exons and encoding a 532 amino acid protein (GenBank accession number AY357073); 2) CERKLb, a 14 exon isoform, which includes an additional exon (4b) between exons 4 and 5 and encodes a 558 amino acid protein (accession number AY690329); 3) CERKLc, a splicing variant devoid of exons 3-4-4b-5, which encodes a 419 amino acid protein (accession number AY690330); and 4) CERKLd, a splicing variant that skips exons 4-4b-5 and encodes a 463 amino acid protein (accession number AY690331). Two very faint bands corresponded to not in-frame splicing variants (accession numbers AY690332 and AY690333) that would generate prematurely truncated proteins. These were considered aberrant transcripts, and given the mRNA quality control regulation, if they were produced in cells, they would be most probably cleared by the nonsense mediated decay mechanism.

The longest isoforms (CERKLa and CERKLb) contained the complete DAG domain and a putative N-terminal pleckstrin homology (PH) region. In contrast, the short forms (CERKLc and CERKLd) skipped most of the DAG kinase domain, although the putative PH domain (encoded in exons 1 and 2) was preserved (Figure 1B).

Noticeably, exons 4b and 5, which are alternatively spliced, show the rare GC donor splice site instead of the more common GT (marked by asterisks in Figure 1A). Both exons and the GC donor site sequences are conserved in the chimpanzee genome. The mouse *Cerkl* gene, which shows overall strong conservation in exon and intron structure, has no equivalent for exon 4b, thus suggesting that there are only 3 CERKL isoforms in the mouse. Again, the donor site of exon 5 in rodents is GC.

### CERKL shows a complex alternative splicing pattern in human tissues

**Figure 2:**
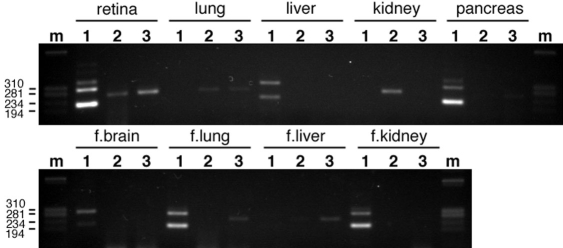
RT–PCR analysis of specific *CERKL* isoforms in several human tissues. For each tissue, lane 1 shows amplification of isoforms a (fast migrating band) and b (slow migrating band), whereas lanes 2 and 3 show the amplified product of isoform c and d, respectively. In the second gel, “f” represents fetal tissue. Marker band sizes are indicated in base pairs (bp). The additional faint bands in lane number 1 correspond to heteroduplexes of the PCR products.

As we had previously found that *CERKL* was expressed in a variety of human adult and fetal tissues [2], we searched for *CERKL* isoforms in tissues other than retina. To this end, we used a panel of first strand cDNAs from several human tissues. In accordance with our previous data, no *CERKL* isoform was found in adult skeletal or heart muscle. However, some of the four in-frame splicing variants were detected in other adult and fetal tissues (Figure 2). cDNAs encoding protein isoforms CERKLa and CERKLb (with an intact DAG kinase domain) were present in adult liver and pancreas, as well as in fetal brain, lung, and kidney. One or both short isoforms (c and d) were detected in adult lung, adult kidney, adult pancreas, fetal lung, and fetal liver. Notably, retina was the only tissue in which all 4 isoforms were expressed under the conditions tested. However, basal levels of most isoforms were observed in most tissues after a saturating number of PCR cycles (data not shown).

### CERKL does not phosphorylate ceramide under standard conditions

**Figure 3:**
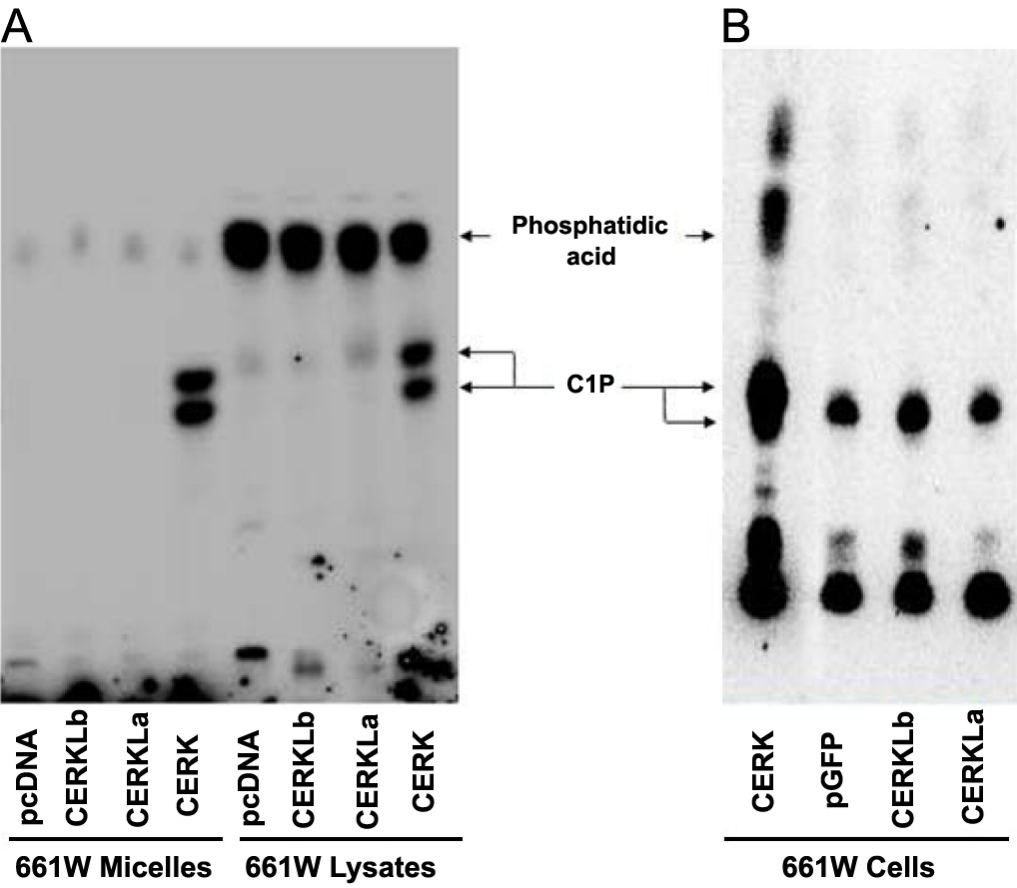
CERKL kinase activity assays. A: The autoradiography of one out of many thin layer chromatographies (TLC) from in vitro assays is shown. Lipid micelles and heat-inactivated cell lysates from 661W (murine photoreceptor-derived cell line) cells were used as substrates, whereas protein lysates from COS-7 cells transfected with either empty vector (pcDNA), CERKL isoform a (532 aa), CERKL isoform b (558 aa) or ceramide kinase CERK (positive control) expressing constructs were the source of the enzymatic activity. **B:** Autoradiography of a TLC from in vivo assays is shown. Cultured 661W cells were transfected with either pGFP (empty vector), or CERKLa-GFP, CERKLb-GFP, or CERK-GFP (positive control), selected by FACS and grown in a medium supplemented with ^32^P-orthophosphate (see Material and Methods for details on the protocols). C1P represents Ceramide-1-phosphate. The images are representative of many different assays, with several experimental parameters, such as the type of cell line and the CERKL isoforms overexpressed, changed. The results of the assays were negative under all the conditions tested.

Given the structural similarities of CERKL to the reported ceramide kinases, a series of experiments were conducted to assess ceramide kinase activity. The protocol from [10] with minor modifications was used for in vitro ceramide kinase assays. We changed either of the following: 1) the source of the ceramides, namely a commercial mixture of brain ceramides (Sigma) or lipid micelles prepared from 661W cells (mouse photoreceptor precursor cell line, kindly provided by M.R. Al-Ubaidi) [11]; or 2) the source of the enzymatic activity to be tested, namely protein lysates from transiently transfected HEK293T and COS-7 cells overexpressing each CERKL isoform, or affinity-purified recombinant GST-CERKL variants (after expression in *E. coli* cells and only used in assays in vitro). No kinase activity was detected for any isoform at any combination of the lipid and protein lysate sources (Figure 3A). Additionally, lysates from co-transfections with constructs expressing 2 different isoforms, or the 4 of them together, were assayed for kinase activity. However, no significant difference with respect cells transfected with the empty vector or single CERKL isoforms could be detected (data not shown). We then attempted to detect CERKL kinase activity in vivo using photoreceptor-derived 661W cells. As 661W are neuronal, they transfect poorly; therefore, we used CERKL-GFP fusion constructs to enrich by FACS the population of transfected cells. Unfortunately, no differences were detected between cells transfected with CERKL and the empty vector (Figure 3B).

The absence of kinase activity prompted us to test the ability of CERKL isoforms to bind sphingolipids by protein-lipid overlay. As PH domains have been reported to have an affinity for phosphoinositides, these substrates were also included. We used the CERKLa isoform (532 aa, containing the complete DAG kinase domain) either as a pure preparation of recombinant GST-CERKL fusion protein (in *E.coli*) or as total fresh protein lysate from HEK293T cultured cells transiently transfected with the CERKLa HA-tagged construct. Again, no positive binding signals were produced (data not shown).

### Subcellular CERKL localization is highly dynamic

Ceramide and other sphingoid lipids are highly dynamic membrane components. Proteins involved in sphingolipid metabolism appear to be specifically localized in subcellular compartments of the secretory pathway, from the ER-Golgi network to the plasma membrane. In this context, we aimed to determine CERKL subcellular localization by transient expression of each alternative splicing variant in cultured COS-7 cells, as the 4 isoforms display a specific domain architecture, which might account for slight differences in function or compartment localization. We used C-terminal HA-tagged CERKL isoforms (CERKL-HA), as well as the fusion CERKL-GFP constructs, in these transient transfections.

**Figure 4:**
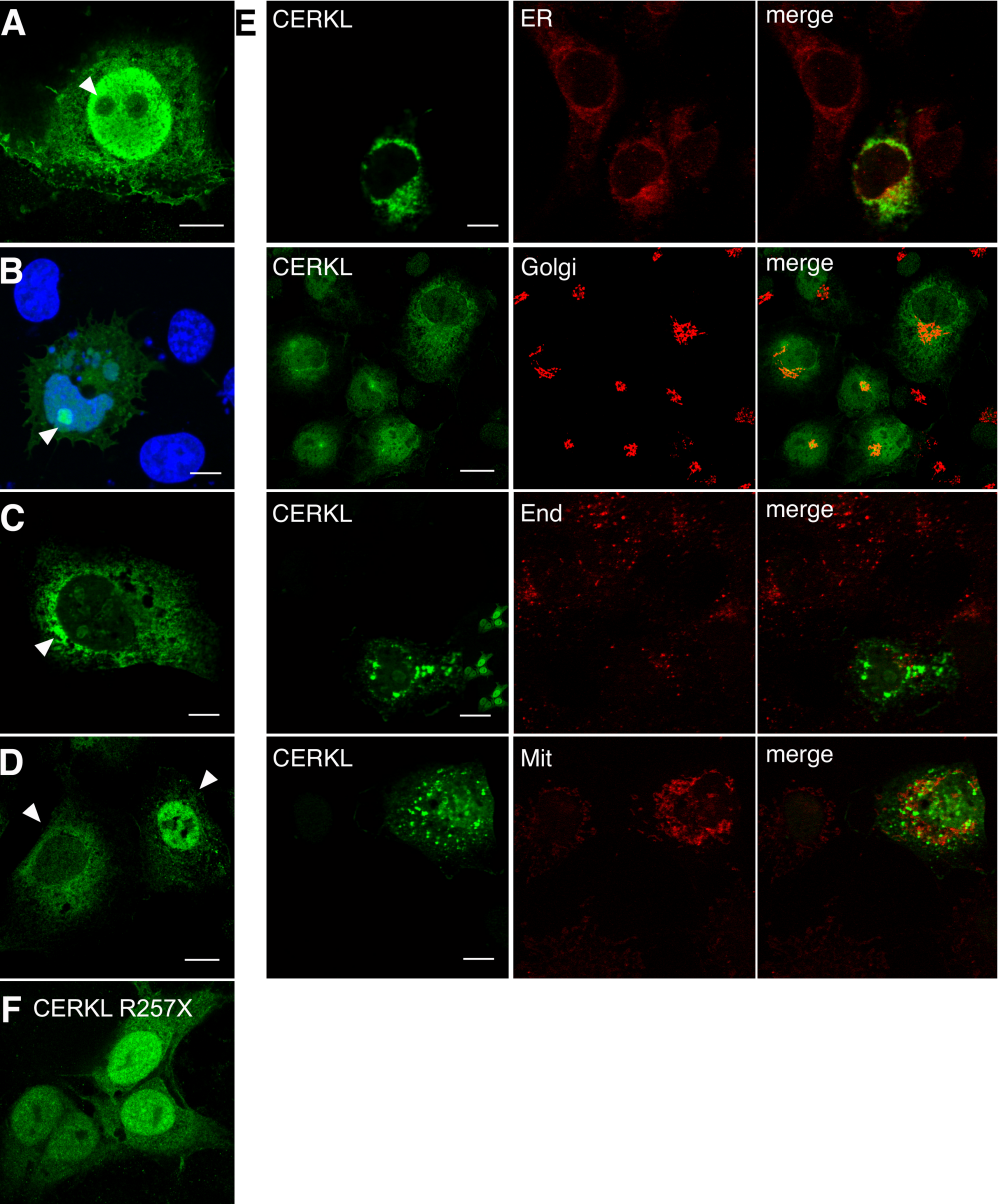
CERKL-HA (isoform a, 532 amino acids) shows a dynamic subcellular localization in COS-7 transfected cells. **A:** Several cells per field showed uniform distribution of CERKL in the cytosol and the nucleus, with clear exclusion from the nucleoli. **B:** A similar pattern but with strong accumulation in the nucleoli (nuclei, counter-stained with DAPI, appear in blue). **C** and **D:** In most cells, CERKL was absent from the nucleus and instead accumulated in clusters, preferentially in the perinuclear region (**A**). These two patterns were cell-specific and could be observed in the same field **(D)**. **E:** CERKL localized to several membranous subcellular compartments, mainly ER and Golgi. The markers used were calnexin for ER, GM130 for Golgi, EEA1 for endosomes and MitoTracker for mitochondria. **F:** R257X truncated CERKL localized preferentially in the nuclei, although it was also detected in the ER. Arrows highlight the relevant CERKL localizations, as immunodetected with an anti-HA monoclonal antibody. Scale bar corresponds to 10 μm. The same results were obtained for all the CERKL isoforms, irrespective of the epitope used, HA or GFP (data not shown). Abbreviations: endoplasmic reticulum (ER), endosomes (End), mitochondria (Mit).

Interestingly, neither a unique localization pattern nor a restricted subcellular localization could be ascribed to any CERKL isoform, as all variants showed similar patterns that appeared to be cell-specific. Two main protein localizations emerged consistently in all preparations, as seen for the isoform CERKLa (532 amino acids; Figure 4A–D). Most cells showed CERKL in a strong perinuclear distribution, while the nucleus was practically devoid of signal (Figure 4C,D). Additionally, few cells in each field presented CERKL within the nucleus, where it was either localized to, or clearly excluded from, the nucleoli (Figure 4A,B,D). No exclusive colocalization with reference markers of subcellular compartments was observed, as CERKL colocalized both with the ER (calnexin) and Golgi matrix (GM130) markers (Figure 4E). To further assess association of CERKL with other membranous compartments, we assayed colocalization to endosomes (EEA1) or mitochondria (labeled by MitoTracker staining). However, no precise colocalization was observed (Figure 4E). Instead, the mutant RP truncated protein CERKLa R257X (the localization of CERKLb 257X was identical; data not shown) showed a more stable localization pattern in the ER and nuclei (Figure 4F), in agreement with other reports [12,13]. Similar CERKL subcellular localization results were obtained at 24 h or 48 h post-transfection, although a shift toward perinuclear accumulation was clearly observed. The same pattern was obtained when analyzing the localization of all 4 CERKL-GFP isoform constructs (data not shown). Overall, these results point to a highly dynamic localization of CERKL, which may relocate according to the stimuli acting upon a cell.

**Figure 5:**
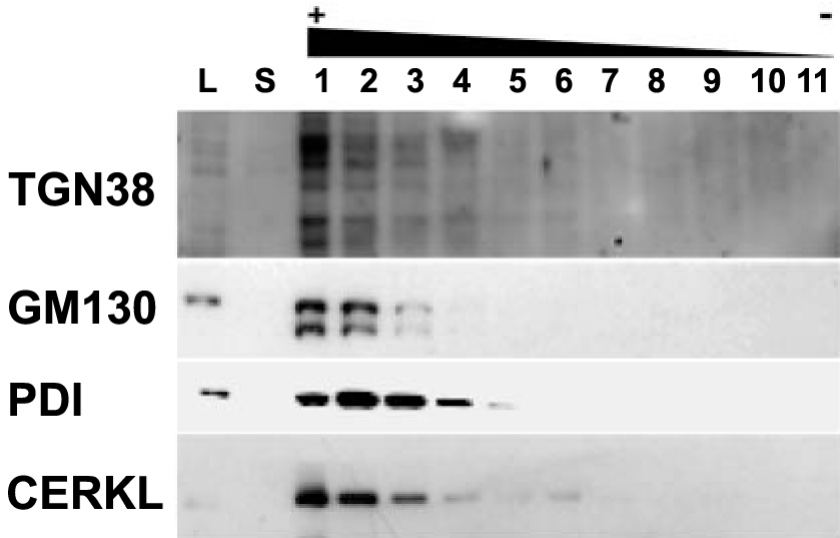
CERKL colocalizes with Trans-Golgi, Golgi and ER markers. HeLa cells transfected with CERKLa-HA were submitted to subcellular fractionation by sucrose gradient. The strongest signal of CERKLa coincides with the Trans-Golgi TGN38 marker, but it is also localized in the same fractions as the Golgi GM130 marker and extends to the ER PDI marker fractions. The multiple banded pattern observed in the TGN38 immunodetection has been previously reported as this protein undergoes complex post-translational modification events. CERKLa-HA was immunodetected with a monoclonal anti-HA antibody. The lanes are numbered according to the sucrose fractions as collected from 1 (bottom, with higher sucrose concentration) to 10 (top, lower sucrose concentration). Abbreviations: total protein lysate (L); supernatant after pelleting the membranes by centrifugation at 100,000x g (S).

To further confirm that CERKL was localized at the membranous organelles, we performed subcellular organelle fractionation in a sucrose gradient. Cells transfected with the wild-type CERKLa isoform were homogenized, and nuclei and cellular debris were discarded. Membranes were pelleted by centrifugation, resuspended in 1.5 M sucrose, and further centrifuged in a 5-step sucrose gradient, ranging from 1.5 M to 0.25 M. Fractions were collected from bottom to top, run in an SDS–PAGE and immunodetected against HA (for CERKL detection) or distinctive organelle markers. The results (Figure 5) clearly showed that CERKL distributed to the same fractions where the Trans-Golgi (TGN38), Golgi (GM130), and ER (PDI) markers localized, although CERKL was more abundant in the Trans-Golgi and Golgi fractions.

### CERKL protects cells from apoptosis in oxidative stress conditions

One of the working hypotheses to explain why mutations in *CERKL* cause RP is based on its cellular protective role through the regulation of the ceramide/ceramide-1-phosphate ratio [14]. Photoreceptors are under constant oxidative challenge. Environmental stress as well as genetic mutations (e.g., on *CERKL*) would make them more susceptible to damage, tilting the survival/death balance and triggering apoptosis. To test whether CERKL has a protective role, we assayed the effect of CERKL overexpression in transiently transfected cells under normal or oxidative stress conditions. As most neuronal cell lines, retina derived cells are very poorly transfected, so we had to resort to transfections on COS-7 and HeLa cell lines to obtain statistically significant results.

**Figure 6:**
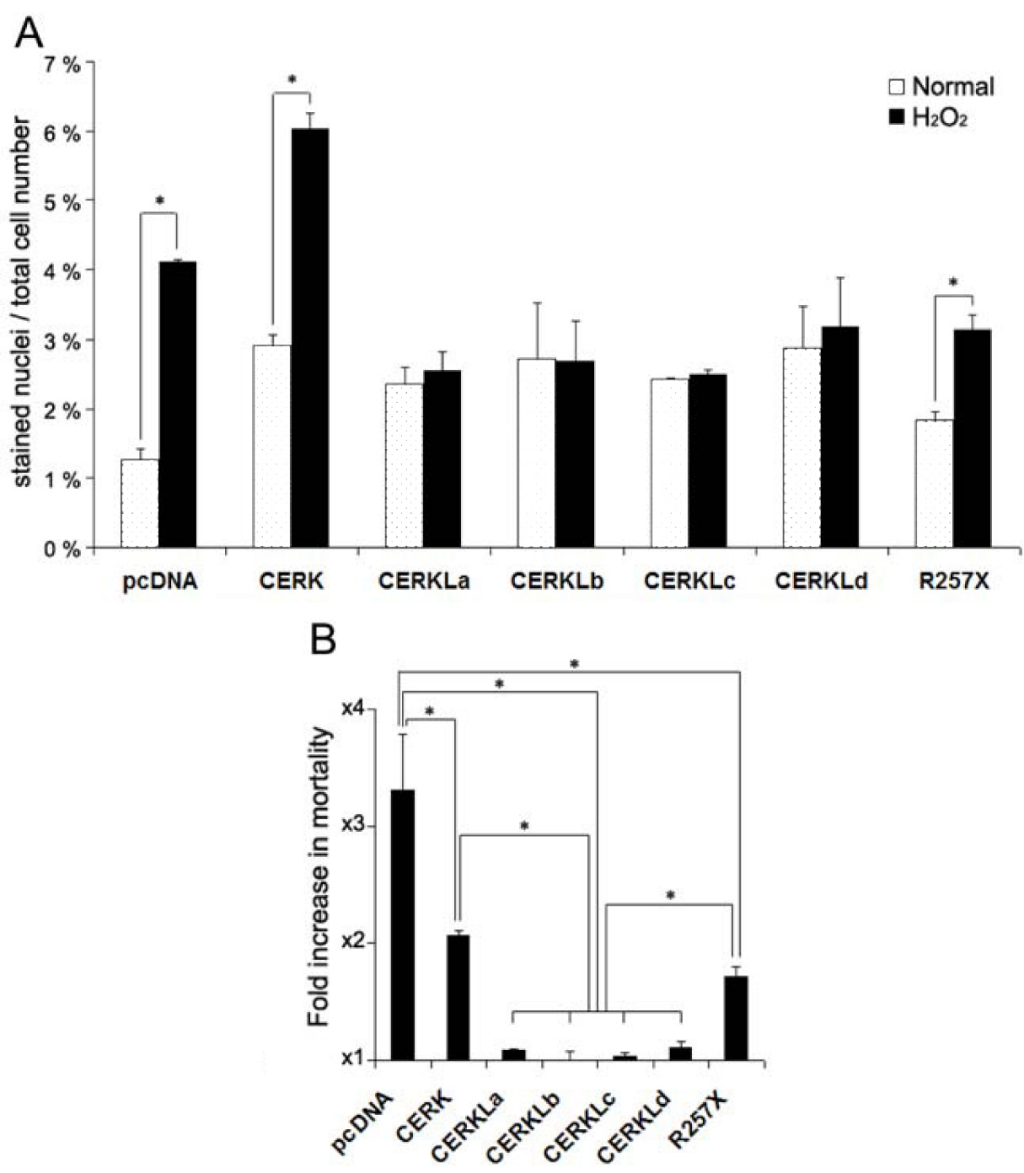
Cells overexpressing CERKL showed no mortality increase in a comparison of normal and oxidative stress conditions. **A:** The histogram shows the mortality (depicted as percentage) of pyknotic nuclei of COS-7 cells. Cells were transfected with either the wild-type CERKL isoforms (named CERKLa to CERKLd), the truncated RP form (R257X), CERK or the empty vector (negative control) and grown in either normal conditions (dotted bars) or treated for 4 h with 200 μM H_2_O_2_ (solid bars). There was no increase in mortality when the cells were transfected with any of the wt CERKL isoforms. In contrast, there was an increase in mortality in cells transfected with, either the empty vector, a CERK (ceramide kinase) construct or the R257X CERKL mutant. Statistical significance is shown by an asterisk (Mann–Whitney test, p<0.05). **B:** The histogram shows the mortality fold-increase of H_2_O_2_-treated versus untreated cells for each construct. Statistically significant differences are shown by asterisks (Mann–Whitney test, p<0.05). More than 500 cells were counted for each construct and condition, in 3 independent replicates.

COS-7 cells were transfected with each of the CERKL isoforms as well as the mutant RP protein, and cell death was assayed by propidium iodide staining after treatment with 200 μM H_2_O_2_ for 4 h. The results were compared to those obtained with cells transfected with either empty vector or a construct expressing the known ceramide kinase, CERK (this construct was a gift from F. Bornancin). Dead cells were stained with propidium iodide and quantified (as a percentage) against total cell number (Figure 6A). Expression of the CERKL or CERK constructs caused a slight increase in mortality in basal conditions. Under oxidative stress, there was a 3.4-fold increase in mortality in the empty-vector transfected cells, whereas cells overexpressing any of the 4 CERKL isoforms maintained a similar mortality rate (no increase in mortality) with a high statistical significance (Mann–Whitney, p<0.05; Figure 6B). Remarkably, the CERKL R257X (the RP mutant) failed to protect cells; the mortality rate increased under oxidative stress (Figure 6B), although to a lesser level than the empty-vector cells (1.8 fold increase in mortality; statistical significance, p<0.05 in comparison to either CERKL isoforms or the empty vector). As propididum iodide staining was performed on coverslips, the transfection efficiency of each construct was assessed by western detection on protein lysates of replicate transfections.

**Figure 7:**
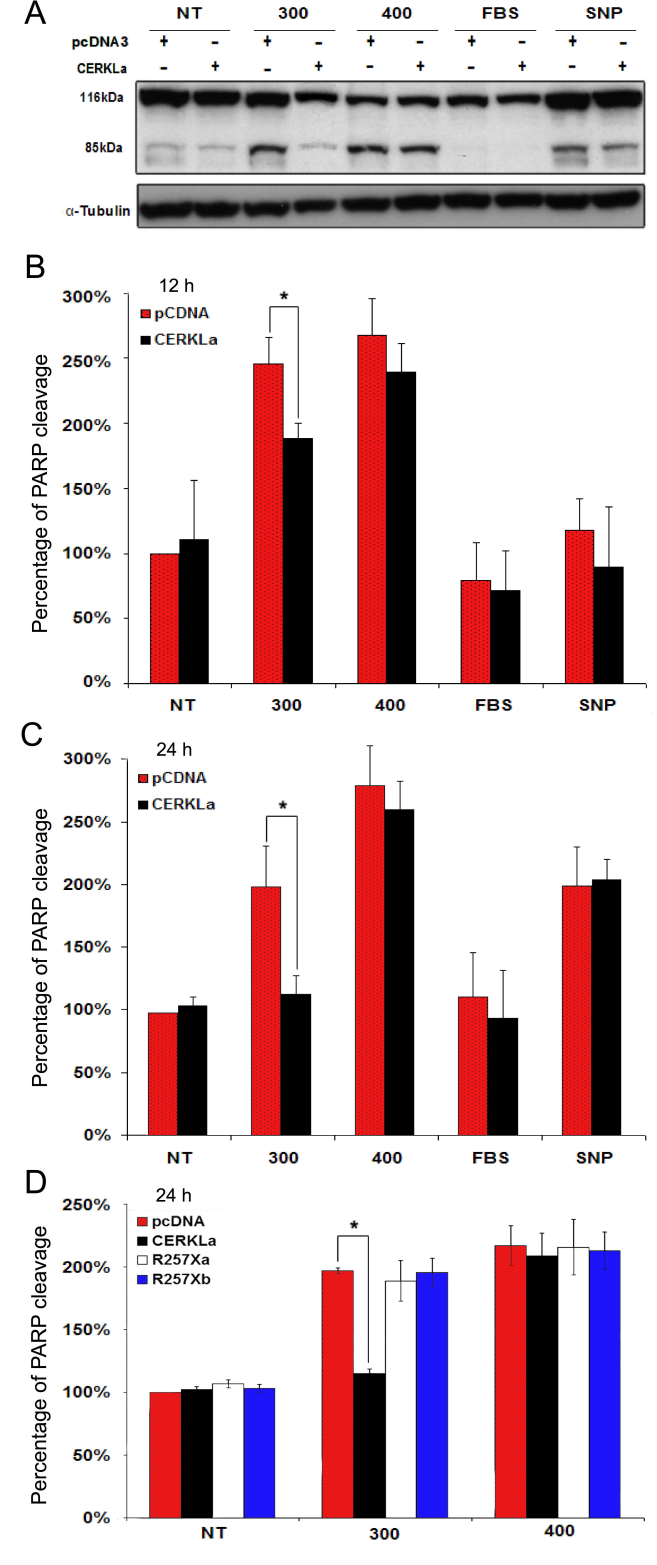
Overexpression of CERKL protects HeLa cells from apoptosis caused by oxidative stress. **A**: The western blot of the PARP apoptosis-dependent cleavage was obtained and immunodetected 24 h after treatment with several oxidative reagents, as indicated. The PARP precursor protein size is 116 kDa, whereas the proteolytic product after caspase-3 cleavage is 85 kDa. Cells were transfected with either the CERKLa (532aa, encompassing the kinasic domain) or the empty vector pcDNA3. Immunodetection of tubulin was used for normalization. This is one image of several similar replicates. The transfection efficiency was comparable as assessed by western immunodetection (data not shown). **B** and **C**: Quantification of the PARP-cleaved peptide with respect to total PARP immunodetected protein (both precursor plus peptide) in empty vector (red bars) versus CERKLa transfected cells (solid bars) under the different treatments. Basal apoptosis in empty vector untreated cells was arbitrarily considered 100%. CERKL protection against apoptosis was clearly detected after 12 h treatment with 300 μM H_2_O_2_ compared with the empty-vector transfected cells, whereas in the other oxidative conditions this protective effect was not significant (**B**). This protective effect of CERKL against apoptosis induced by 300 μM H_2_O_2_ was much more pronounced after 24 h treatment (**C**). **D**: The histogram shows the quantification of the PARP-cleaved peptide with respect to total PARP immunodetected protein (both precursor plus peptide) in empty vector-transfected cells (red bars) versus cells transfected with either CERKLa (solid bars), the R257X CERKLa mutant (white bars) or the R257X CERKLb mutant (blue bars). Cells were grown under normal conditions or treated with different concentrations of H_2_O_2_. Basal apoptosis in empty vector untreated cells was arbitrarily considered 100%. The protective effect is clearly detected for the full-length protein but not for the truncated mutants. NT-untreated cells; 300-cells treated with 300 μM H_2_O_2_; 400-cells treated with 400 μM H_2_O_2_; FBS-cells grown in medium deprived of fetal bovine serum; SNP-cells grown in medium supplemented with 0.3 mM sodium nitroprusside; R257Xa- cells transfected with the construct bearing the R257X mutation in the CERKLa sequence background; R257Xb-cells transfected with the construct bearing the R257X mutation in the CERKLb sequence background. At least 3 independent experiments were used for replication. Statistical significance is indicated by an asterisk (Mann–Whitney test, p<0.05).

Propidium iodide labels both apoptotic and necrotic cell nuclei. We attempted then to determine whether this protective effect of CERKL overexpression was on preventing cells from entering apoptosis. Activation of caspases is a well established event in apoptosis, and detection of caspase-cleaved substrates, such as PARP-1 (poly-ADP-ribose-polymerase)-1, is one of the most widespread hallmarks to quantify cell apoptosis [15,16]. PARP caspase-dependent cleavage was immunodetected in HeLa cells that were transiently transfected with either the empty vector or the CERKLa isoform (532 amino acids, encompassing the DAG kinase domain) in normal or under oxidative stress conditions, and using several reagents and concentrations: 300 μM and 400 μM H_2_O_2_, fetal bovine serum (FBS) deprivation and 0.3 mM sodium nitroprusside (SNP). The analysis was performed at different window times to determine the best range of conditions for quantification of any potential protective effect. CERKL did protect against apoptosis induced by oxidative stress (Figure 7A) in cells treated with 300 μM H_2_O_2_. This effect was dependent on the degree of oxidative insult, as it was much more effective at 300 μM (no apoptosis increase), than at 400 μM H_2_O_2_ (no significant protection). It was also dependent on the type of inducer, as cells overexpressing CERKLa did not behave differently from empty-vector transfected cells after FBS deprivation or SNP addition.

This protective effect was also dependent on time, and presented an effective window frame. It was already detectable at 12 h (Figure 7B) with an average of 20% protection (statistical significance p<0.05, Mann–Whitney test). In addition, this effect appeared to be much more prominent at 24 h (Figure 7C), where the protection appeared to be maximal, around 50% protection (p<0.05, Mann–Whitney test). However, the effect was no longer observed at 48 h (data not shown), where PARP cleavage was high and comparable between empty-vector and CERKLa transfected cells. The expression of CERKLa was comparable in all transfections (65%–68%), as assayed by western blot (data not shown), thus the observed differences were solely attributable to the overexpression of this isoform. Interestingly, this protective effect at 24 h-treatment was only found for the full-length CERKLa but not the truncated R257X mutant (neither in the a nor the b isoform constructs; Figure 7D), in accordance to the pathogenicity of this mutation.

## Discussion

There is mounting evidence that most human genes show alternatively spliced isoforms, many of them regulated by tissue-specific factors. Indeed, alteration of alternative splicing is frequently linked to severe genetic diseases [17]. We report the characterization of 4 in-frame alternatively spliced isoforms of *CERKL*, which is responsible for an autosomal recessive form of retinitis pigmentosa (RP26). In our case, the isoform multiplicity increased the complexity of the functional analysis of a yet to be characterized lipid kinase. For instance, comparison of isoforms CERKLa and CERKLb revealed an additional exon (E4b), which introduces 26 additional amino acids in-frame within the DAG kinase catalytic domain and immediately upstream of the phosphate-donor binding site (GGDGS). The position of this exon in the presumptive catalytic domain makes it a good candidate for modulating the catalytic, substrate- or partner-binding abilities of the protein, as has been postulated for alternatively-spliced exons within encoded functional domains after an in silico genomic survey [18]. Interestingly, the other two in-frame isoforms, CERKLc and CERKLd, lack the exons encoding the DAG kinase (mainly encoded in exons 4 and 5) domain, which may severely compromise the catalytic activity while preserving other functions, among them substrate recognition. That exons 4b and 5 share a rare splice donor site (GC) strongly suggests that the production of these alternatively spliced isoforms is regulated. Genome-wide analyses on the usage of splice sites showed that only 0.69% of exon/intron donor sites are GC [19], making it highly improbable that 2 such sites flanking exons tandemly arrayed occurred in the same gene unless there was some kind of regulation behind. Given that GC- splice donor sites are intrinsically weak, explaining why 60% of the GC-flanked exons are alternatively processed [20], and that this site is evolutionarily conserved, *CERKL* splicing is probably highly regulated.

From sequence comparisons and phylogenetic analysis, CERKL unambiguously clusters within the ceramide kinase subfamily of lipid kinases [2]. However, at present, there is no experimental evidence to support CERKL kinase activity on ceramides. Therefore, it still stands as an orphan lipid kinase, in agreement with other reports [12,13]. It may well be that, although not yet identified for other lipid kinases, a partner is required for substrate recognition or catalysis. Alternatively, the substrate may be a ceramide-derived lipid undetectable under standard ceramide separation conditions. In any case, the presence of 4 different isoforms, of which 2 are devoid of the kinase domain, suggests that CERKL is involved in cellular roles other than lipid phosphorylation. Remarkably, in contrast to other lipid enzyme genes that are spread throughout all the eukaryotes, *CERKL* may be exclusive to the vertebrate lineage. Highly homologous sequences can be found in chick and fish, but no evidence of *CERKL* is found in the lancelet, the closest chordate ancestor to the vertebrates, which instead shows an unambiguous hit to *CERK* (data not shown). Further support to its vertebrate confinement comes from the fact that other invertebrate genomes, such as *Drosophila* and *C. elegans*, are also devoid of any *CERKL* homologs. In this evolutionary context, the more complex vertebrate retina may have required genes with novel enzymatic functions to protect cells against stress, adding further refinement to the metabolism of bioactive lipids as second messengers.

The versatile subcellular localization of CERKL in the COS-7 and HEK293T cell lines is common to all isoforms. This complex and highly dynamic pattern may reflect changes in the intracellular sphingolipid pools, as it occurs with other proteins in the ceramide metabolism [21–24]. CERKL has been reported to contain one bona fide nuclear localization signal (amino acids 102–106) [13], which would account for its import into the nuclei. In our studies and for all wild-type CERKL isoforms, nuclear localization is not a general event. Thus, the evident retention of the truncated R257X protein mutant seemed relevant to pathogenesis [13,14]. Further investigation is needed to determine how and why CERKL is imported into the nuclei and the nucleoli as well as what is the function of the protein in these organelles. Indeed, transient nuclear localization of CERKL may be related to the regulation of the chromatin-associated ceramide and sphingomyelin pools, in accordance with the intranuclear distribution of other key enzymes of the ceramide metabolism, as happens for SMase [25], ceramidase [26], and SPHK2 [27]. In addition, the consistent localization in the ER and Golgi compartments is compatible with the membrane-associated roles—such as non-vesicular trafficking—reported for lipid kinases and other lipid-related proteins. It has been claimed that compartmentalization of ceramide function requires transfer between different membranous organelles [8,21,23], although ceramide kinase activity has also been detected in the cytosol, where it is not associated with membranes [10], This indicates, overall, that these enzymes translocate between different compartments. In this scenario, the observed variable CERKL intracellular distribution is not so surprising, particularly when considering that similar protein localization dynamics—which shift depending on the cellular state or stimuli—are now being reported for other proteins involved in lipid metabolism [5,24,28]. Under our conditions, the 4 isoforms showed a similar localization pattern, irrespectively of the epitope used (HA or GFP), suggesting that the functional differences among them are not dependent on subcellular localization. Besides lipid phosphorylation, CERKL isoforms may be involved in the regulation of its own enzymatic activity, lipid trafficking and storage, as well as in binding to distinct substrates or protein partners. In this context, the multiplicity of isoforms in the same tissue (or even in the same cell) and the fact that two of the four isoforms lack the lipid kinase domain, gives support to the rationale of multiplicity of functions.

RP is characterized by progressive depletion of photoreceptor cells through apoptosis. Many different genetic defects converge in retinal cell malfunction and trigger programmed cell death. The retina consists of several ordered layers of differentiated neuronal cells that are under constant oxidative stress conditions due to light exposure and a high metabolic rate. Therefore, photoreceptor survival depends on the action of anti-apoptotic mechanisms to prevent premature death. Our data support that CERKL plays a crucial protective role as its overexpression protects cells from entering apoptosis. This protective effect is dependent on time and the severity of the insult suggesting that CERKL overexpression is effective only under a range of stress conditions, but probably not after massive cell damage. Remarkably, the R257X protein causing RP cannot protect cells after sustained injury. The weak protective effect observed at 4 h treatment with H_2_O_2_ was no longer detectable after 24 h treatment. This RP mutation lies within the *CERKL* exon 5, which is skipped in the variants that do not contain the DAG kinase domain (isoforms CERKLc and CERKLd). Thus, these two variants probably are not compromised in the RP26 patients. In this context, the physiologic relevance of each CERKL isoform and their particular contribution to retinal disorders is yet to be determined. Conceivably, the RP pathology could be caused by the absence of the longer isoforms or alternatively, the toxicity of the 2 truncated proteins. Our results, and the fact that most transcripts with premature STOP codons are degraded by nonsense mediated decay, support the first scenario.

To our knowledge this is the first time CERKL has been shown to be directly involved in protection against apoptosis. Thus, we link RP-causative mutations to a regulatory mechanism for cell survival, which highlights the feasibility of an RP-gene therapy approach based on protection factors to prevent photoreceptor neurodegeneration and promote cell survival.
